# Unfunctionalized and Functionalized Multiwalled Carbon Nanotubes/Polyamide Nanocomposites as Selective-Layer Polysulfone Membranes

**DOI:** 10.3390/polym14081544

**Published:** 2022-04-11

**Authors:** Seham S. Alterary, Raya M. Alyabes, Ahmed A. Alshahrani, Monirah A. Al-Alshaikh

**Affiliations:** 1 Chemistry Department, College of Science, King Saud University, P.O. Box. 2455, Riyadh 11495, Saudi Arabia; 439203058@student.ksu.edu.sa (R.M.A.); mshaikh@ksu.edu.sa (M.A.A.-A.); 2 National Centre for Radioactive Waste Treatment, King Abdul Aziz City for Science and Technology, Riyadh 11442, Saudi Arabia

**Keywords:** polyamide, multiwalled carbon nanotubes, membrane, polysulfone, desalination

## Abstract

Nowadays, reverse osmosis is the most widely utilized strategy in membrane technology due to its continuous improvement. Recent studies have highlighted the importance of the surface characteristics of support layers in thin-film membranes to improve their reverse osmosis performance. In this study, interfacial polymerization was used to generate the membranes by employing polyamide as a selective layer on top of the polysulfone supporting sheet. Different membranes, varying in terms of the concentrations of unfunctionalized and functionalized multiwalled carbon nanotubes (MWCNTs), as well as ethanol, have been fabricated. The efficiency of the membrane has been increased by increasing its permeability towards water with high salt rejection. Different characterization techniques were applied to examine all of the fabricated membranes. PA-EtOH 30% (*v*/*v*), as a selective layer on polysulfone sheets to enhance the membrane’s salt rejection, was shown to be the most efficient of the suggested membranes, improving the membrane’s salt rejection. The water permeability of the polyamide membrane with EtOH 30% (*v*/*v*) was 56.18 L/m^2^ h bar, which was more than twice the average permeability of the polyamide membrane (23.63 L/m^2^ h bar). The salt rejection was also improved (from 97.73% for NaCl to 99.29% and from 97.39% for MgSO_4_ to 99.62% in the same condition). The PA-MWCNTs 0.15% membrane, on the other hand, had a reduced surface roughness, higher hydrophobicity, and higher water contact angle readings, according to SEM. These characteristics led to the lowest salt rejection, resulting from the hydrophobic nature of MWCNTs.

## 1. Introduction

Oceans, seas, rivers, lakes, and underground streams account for over 70% of the world’s water reservoirs [1]. Fresh water accounts for around 2.5% of the Earth’s total water capacity, according to indicators from the United Nations Water Statistics. Water scarcity, defined as a high rate of usage compared to the available supply, has been created by a variety of factors. Some elements are caused by natural events, while others are generated by human activity, both of which have the potential to change the physical environment [2]. As a result of overpopulation, urbanization, and industrial growth, global warming has an impact on the availability of water supplies. Many sectors are developing to meet the demands of population growth, putting excessive demands on water and causing the depletion of water and its resources [3]. The phrase “water purification” refers to any unit’s operation and processes that entail techniques and subsequent stages for the removal of harmful impurities. Physical, chemical, biological, or radioactive pollutants may be present. Membrane processes are involved in the production of clean water, wastewater treatment/purification, and water recycling around 53% of the time [4]. Membrane separation is one of the most successful, convenient, and promising techniques used in water treatment systems [5]. When driving forces are applied to membrane water treatment, pollutants must be removed from the water [6]. A pressure difference is the most common driving force in membrane separation [7]. Membrane technologies used in water purification include reverse osmosis (RO), nanofiltration (NF), microfiltration (MF), and ultrafiltration (UF). Reverse osmosis (RO) is a better tool and system that allows several purifying jobs to be completed in one operation. RO membranes are capable of effectively removing salts and contaminants. A membrane separation procedure separates the water from the solutes in a pressured saline solution without phase change or heating [8]. Furthermore, it has little impact on the product pH or chemical alterations. The water is purified using a RO membrane, which can remove nearly all soluble particles from the water and fulfills the direct drinking standard [9]. The two most popular types of primary RO membrane are cellulose acetate (CA) and aromatic polyamide (PA) [10,11]. A thin-film composite polyamide membrane generated by interfacial polymerization of m-phenylenediamine (MPD) and trimesoyl chloride (TMC) over a porous polysulfone (PS) substrate is the most commercially successful membrane [12]. PA thin-film composite membranes have a stable structure, low environmental demands, less biological pollution, pressure resistance, and a wide range of applications. In thin-film composite membranes, a thin active layer is frequently placed on top of a significant support layer [13,14]. Polymeric membranes should ideally be hydrophilic, chemically resistant, and microbially resistant, as well as physically, thermally, and structurally robust over lengthy periods of operation [15]. The inherent balance between membrane selectivity and permeability is a key difficulty in membrane technology [16].

Carbon nanotubes (CNTs) exhibit the promised qualities for the purification of water, including membrane deterioration via bacterial growth suppression and separation selectivity improvement due to their narrow pore size distribution [17]. CNTs have a large specific surface area, as well as a large number of adsorption sites and variable surface chemistry [18]. The MWCNTs functionalization technique also averts agglomeration and ameliorates interfacial adherence between the nanotubes’ graphitic sidewalls and the polymer [19]. Shawky et al. [20] developed nanocomposite-based-multiwalled carbon nanotubes/polyamide with 3-fold higher NaCl rejection capacity than a pure polyamide membrane, according to the literature review. The flow rate of the polysulfone (PS) membrane also increased when the loading percentage of multiwalled carbon nanotubes increased. Because of its increased activities, such as high-water permeability with salt rejection, the fabrication of nanocomposite membranes for water desalination has recently received a lot of attention. There has been significant progress in the use of nanomaterials for filtration-based desalination processes. Fahmey et al. [21] described a comparative evaluation of nanomaterials’ mixed polysulfone effect for enhanced the membrane distillation for water desalination. Das et al. [22] reported a prospective overview on the effect of particle size on the toxicity of CNTs and the purification of water. Another study performed by Das et al. [23] described the impact effect of functionalization of MWCNTs on their solubility in water and acetone.

The goal of this study was to improve RO membranes constructed of carbon nanotubes (MWCNT) and polyamide (PA) via interfacial polymerization of m-phenylenediamine (MPD) and trimesoyl chloride (TMC) on a polysulfone (PS) membrane for use in the desalination process. The RO membranes’ surface shape, mechanical characteristics, hydrophobicity, and thermal stability were all studied. This research also focused on water permeability, the rejection performance of RO membranes to inorganic electrolytes, and the effect of EtOH on membrane performance.

## 2. Materials and Methods

### 2.1. Materials

Polysulfone membrane sheet, PS (PS-20), (Sepro, California, CA, USA). Merck supplied 1,3-phenylenediamine (C_6_H_8_N_2_, 99.5%) and 1,3,5-benzenetricarbonyl trichloride (C_9_H_3_Cl_3_O_3_, 98%) (Kenilworth, New Jersey, NJ, USA). Short multiwalled carbon nanotubes (MWCNTs) with a purity of 95% and average diameter of 3–10 nm was provided by Grafen Chemical Industries (Ankara, Turkey). MWCNTs COOH and MWCNTs-NH_2_) are thin multiwalled carbon nanotubes that have been functionalized. MWCNT samples with a purity of 95% were obtained from Nanocyl (Sambreville, Belgium). Hexane (C_6_H_14_, 99%) was purchased from Oxford Laboratory (Mumbai, India). Sodium chloride (NaCl, 99%) was purchased from Sigma-Aldrich Chemicals (Hamburg, Germany), while anhydrous magnesium sulfate (MgSO_4_, 98%) and extra pure anhydrous sodium carbonate (Na_2_CO_3_, 99.5%) were obtained from Scharlau (Barcelona, Spain). Absolute ethanol (96%) was purchased from Ajax Finechem Pty, Ltd. (South Wales, Australia). Milli-Q^®^ water (18.2) was used to make both solutions and dilutions in this study.

### 2.2. Preparation of Membrane 

#### 2.2.1. Polyamide Membrane

**Figure 1:**
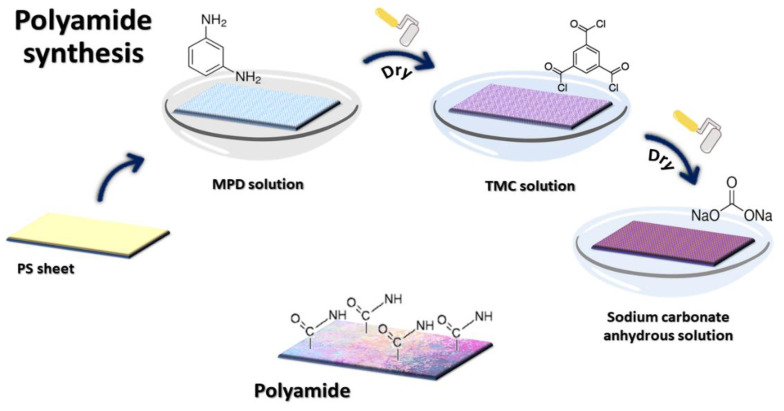
Diagram of PA membrane preparation.

The fabrication of the RO membrane (Figure 1) was conducted in four steps. The first step involved the modification of the PS membrane support layer. The MPD solution 2% (*w*/*v*) was prepared in the second step. The preparation of a 0.1% TMC solution (*w*/*v*) was carried out in the third step. The final step was accomplished by preparing an anhydrous sodium carbonate solution of 2% (*w*/*v*). 

A polyamide layer on top of PS was used to conduct interfacial polymerization (IP) between the MPD and TMC supporting membrane sheets. The PS support sheets were adjusted before beginning by soaking them in an aqueous solution for 24 h at room temperature. After that, the PS sheet was immersed in an aqueous MPD solution for 2 min. The excess MPD was removed with a rubber roller. After 1 min in the TMC solution (0.1% *w*/*v*), the substrate membranes were rolled over the support surface with a rubber roller to remove surplus solution. Finally, they were washed for 10 min with a 2% (*w*/*v*) anhydrous sodium carbonate solution before being kept in distilled water [24].

#### 2.2.2. PA-MWCNT Membranes

PA-MWCNT membranes were made using the same procedure as that described in Section 2.2.1. Briefly, PA-MWCNT dispersions were prepared by mixing 15 mL of MPD with MWCNTs at 0.08% (*w*/*v*), 0.1% (*w*/*v*), or 0.15% (*w*/*v*), 0.08% MWCNTs−COOH (*w*/*v*), and 0.08% MWCNTs–NH_2_ (*w*/*v*) in an aqueous solution. The MWCNTs were then dispersed in the solution using sonication at various intervals to maximize their dispersion. Sonication was performed with a 30 W power output and 0.5 s on/off pulses. To prevent temperature spikes during sonication, the sample bottle was placed in a water bath.

#### 2.2.3. PA-EtOH

Before 1 h of work, PS sheets were immersed in different concentrations of ethanol: 10% (*v*/*v*), 20% (*v*/*v*), or 30% (*v*/*v*). The procedure was completed as previously described in Section 2.2.1.

### 2.3. Characterization and Evaluation

Various instrumentation methods were employed to confirm and assess the membranes. 

#### 2.3.1. Sonication Time 

To verify that the observed absorption was inside the instrumental optimum range, Milli-Q water was used to dilute the dispersion solutions (PA-MWCNTs, MWCNTs-COOH, and MWCNTs-NH_2_). The UV-Vis-NIR spectra of each dispersion solution were measured in a quartz cuvette at room temperature (about 21 °C). A Cary^®^ 500 UV-Vis-NIR spectrophotometer (Llantrisant, UK) was applied to measure the absorption maxima at 300–800 nm for all suspended solutions. To determine the membrane surface’s hydrophilicity/hydrophobicity, the water contact angle was estimated using the sessile drop method. Two liters of Milli-Q were progressively deposited over the drier membrane surface using a microsyringe. Measurements were taken a minimum of five times for each membrane, and the mean average was determined.

#### 2.3.2. Thermal Stability 

A PerkinElmer analyzer was used to perform the thermogravimetric analysis (TGA) of the membranes (Massachusetts, MA, USA). The TGA experiment was carried out in the presence of nitrogen gas at a temperature range of ambient temperature to 800 °C with a heating rate of 10 °C/min.

#### 2.3.3. Zeta Potential (ZP)

The zeta potential (ZP) of all membrane surfaces was monitored using a SurPASS electrokinetic analyzer (Anton-Paar GmbH, Graz, Austria). At room temperature, all zeta potentials of the membrane were acquired with a 1 mM KCl supporting electrolyte solution. To modify the KCl solution, a 0.05 M HCl solution was utilized, and pH values ranging from 2.5 to 5.5 were calculated for all automatic titration measurements.

#### 2.3.4. Scanning Electron Microscope (SEM)

Images of the top surfaces, as well as cross sections, of membranes were obtained using an SEM-JSM-7610F (JEOL Ltd., Tokyo, Japan). To boost the conductivity and improve imaging, the membrane samples were dried for 24 h at ambient temperature before being coated with platinum.

#### 2.3.5. Salt Rejection of Water 

To describe all membranes with an area of 40 cm^2^, the water flux and salt rejection were measured. A salt solution combining sodium chloride (NaCl) and magnesium sulfate (MgSO_4_) at a concentration of 2 g/L was used as a feed solution; each salt was individually evaluated. Water fluxes and salt rejection were investigated at pressures ranging from 6 to 25 bar, with a pH of 7. All data on water flow and salt rejection were taken after being compressed for about 45 min at 28 bar pressure to produce a steady-state functioning. All of the tests were performed at ambient temperature (21 °C 2 °C) with Milli-Q^®^ water.

The water flux was calculated using Equation (1):

(1) $$J=\frac{V}{A\times t},$$

where *J* is the water flux (L/m^2^ h), *V* is the permeate volume (L), *A* is the membrane area (m^2^), and *t* is the treatment time (h).

The salt rejection (*R*) was calculated using Equation (2):

(2) $$R\%=\left(1-\frac{C_{P}}{C_{f}}\right)\times 100,$$

where *C_p_* and *C_f_* are the salt concentration in the permeate and feed streams, respectively.

## 3. Results

### 3.1. Optimization of Sonication Time

**Figure 2:**
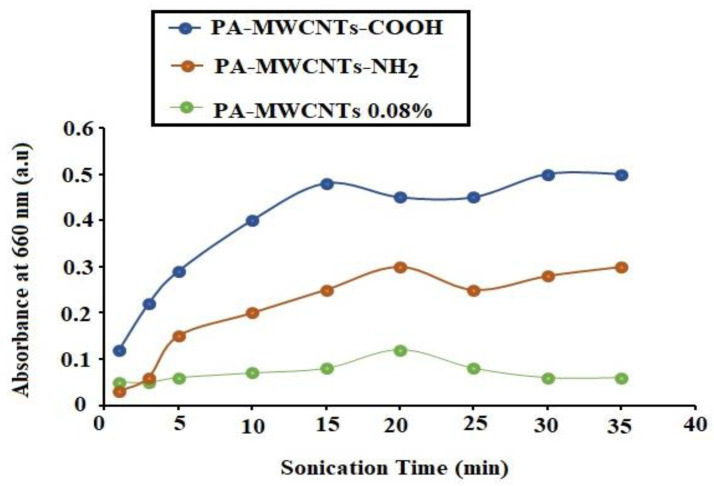
Sonication time of PA-MCWNTs, PA-MWCNTs−COOH, and PA-MWCNTs-NH_2_.

PA-MWCNTs, PA-MWCNTs-COOH, and PA-MWCNTs-NH_2_ were all subjected to sonication. The three dispersions’ UV-Vis-NIR spectra (300–800 nm) were investigated. By extending the sonication period, the absorbance at 660 nm was dramatically increased [25]. As shown in Figure 2, the UV-Vis absorbance of each of the three dispersions at 660 nm was plotted versus sonication time. The results showed that 20 min of sonication was enough to disperse the three dispersion solutions (MWCNTs, MWCNTs-COOH, and MWCNTs-NH_2_) in the PA solution.

After 20 min, there were no remarkable difference in the absorption of the three dispersions at 660 nm. It should also be mentioned that the three investigated solutions (PA-MWCNTs, PA-MWCNTs-COOH, and PA-MWCNTs-NH_2_) were comparable to those reported previously with MWCNTs/chitosan and MWCNTs/chitosan-crosslinked [19]. Previous research showed that CNTs would not be distributed for a long time due to the lengthy ultrasonic energy exposure, which would increase flaws and diminish the membrane porosity. This might result in poor mechanical and electrical characteristics, lowering the permeability of nanotube carbon membranes [19].

### 3.2. Thermogravimetric Analysis (TGA)

**Figure 3:**
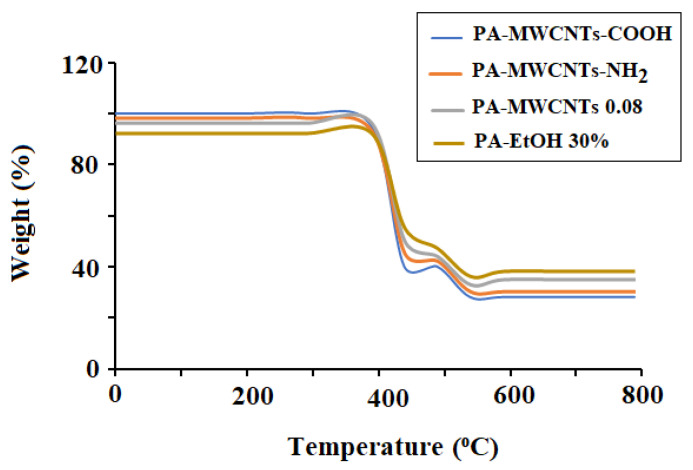
TGA PA-MWCNTs, PA-MWCNTs−COOH and PA-MWCN.

Thermogravimetric analysis has proven to be a useful tool for determining the thermal stability of polymeric systems. The thermal stability of the produced membranes using PA-MWCNTs, PA-MWCNTs-COOH, PA-MWCNTs-NH_2_, and PA-EtOH on a polysulfone support was evaluated using thermogravimetric analysis. Figure 3 clearly shows that the thermal decomposition of all membranes occurred in two stages: the first decomposition step, which resulted in a 59–60% weight loss at temperatures between 375 and 475 °C, and the second decomposition stage, which occurred at temperatures between 475 °C and 700 °C. Surprisingly, a slight degradation phase of 3% weight loss was seen in PS at the 100–170 °C temperature range. This is attributed to the absorbed water removal. PS was the only one that showed a decline phase. It was not found in any other membranes. Because the peaks were formed via sulfone base and amide linkage, the created MWCNTs and EtOH membranes were more stable than PS [26]. PS was stable up to 400 °C. The threshed decomposition temperature provided an indication of the highest processing temperature that can be adopted.

### 3.3. Determination of Zeta Potential (ZP)

**Figure 4:**
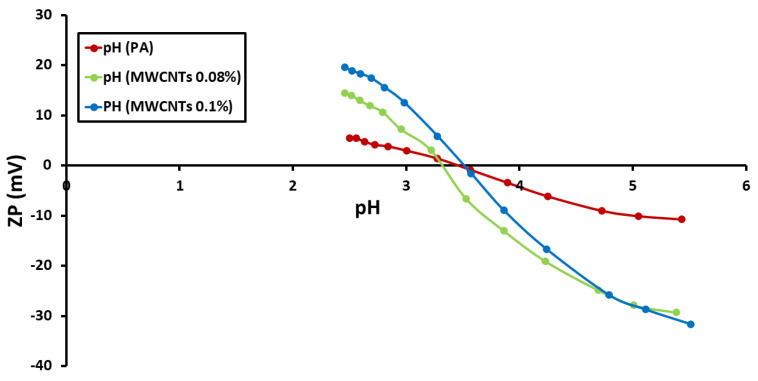
Zeta potentials of PA and PA-MWCNTs (0.08%, 0.1% and 0.15%) membranes as a function of pH.

**Figure 5:**
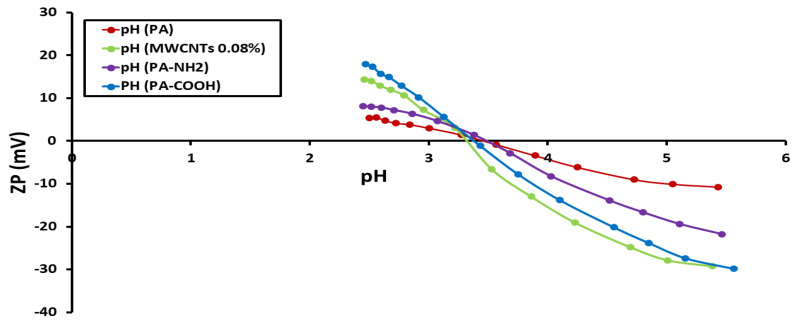
Zeta potentials of PA and PA-MWCNTs, PA-MWCNTs-COOH, and PA-MWCNTs-NH_2_ membranes as a function of pH.

**Figure 6:**
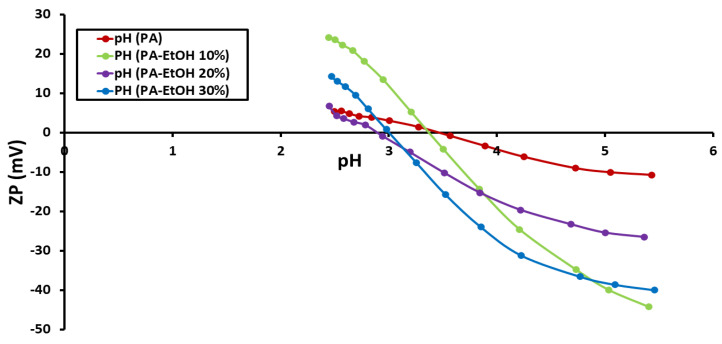
Zeta potentials of PA and PA and PA-EtOH (10%, 20%, and 30%) membranes as a function of pH.

The ZP of each of the nine membrane surfaces was calculated and compared to the pH of the feed solution. Figure 4, Figure 5 and Figure 6 show the ZP measurements for nine produced membranes. The produced membrane has a pattern that is comparable to that of previously described polyamide composites in acidic media. The observed zeta potential variation began at pH 4 [19,27] and, when the pH climbed, the ZP began to have negative values. A more negative membrane was shown to reject more salt due to a coulombic interaction between the negatively charged membrane surface and charged solutes in previous studies. The deprotonation of carboxylic acid groups is to blame for this. Due to the protonation of amine groups, the membranes displayed positive surface zeta potential values at low pH values (3) as well. The positive ZP values at low pH suggested that decreasing the pH value could increase the NaCl adsorption [19,28]. Moreover, the streaming potential observations demonstrated double layer compression as the ionic strength increased and charge neutralization as the pH decreased [29]. The point-of-zero-charge for PA, PA-MWCNTs, PA-MWNCTs-COOH, and PA-MWCNTs-NH_2_ was nearly the same because of the heterogeneous nature of the polyamide membrane. This might be because the membranes include carboxylic acid, amine, and amide functional groups, all of which contribute with titrations [29]. 

### 3.4. Scanning Electron Microscope Analysis (SEM)

**Figure 7:**
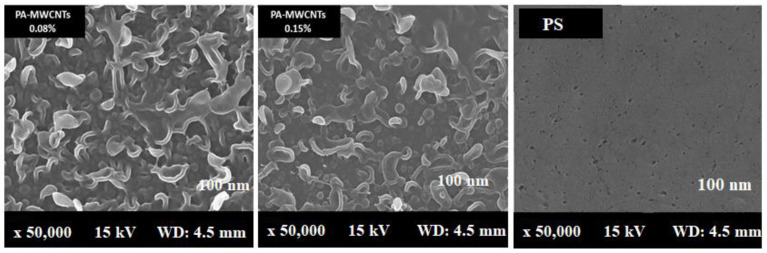
SEM images of the surfaces PA, PA-MWCNTs, and PS membranes.

**Figure 8:**
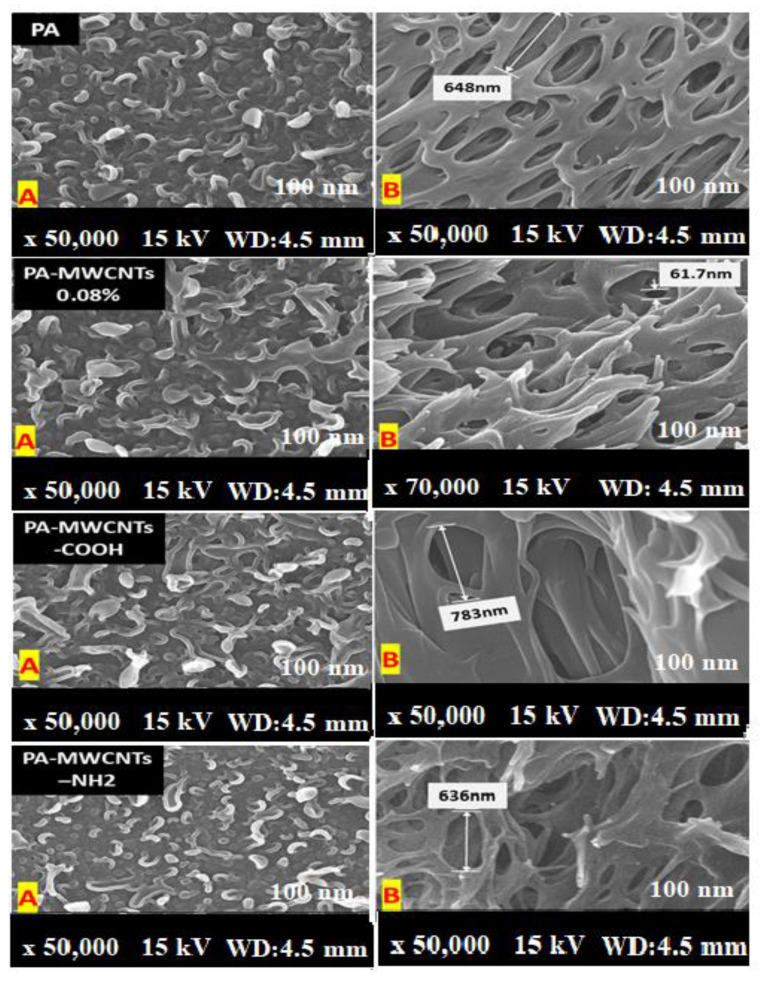
SEM images of the surfaces (**A**) and cross sections (**B**) of PA, PA-MWCNTs 0.08%, PA-MWCNTs 0.08%, PA-MWCNTs-COOH, and PA-MWCNTs-NH_2_.

Because of the interaction between the carboxylic group on the MWCNT walls and the functional groups on the PA chain, SEM images of the surface and cross section of PA-MWCNTs-COOH and -NH_2_ showed that they were better disseminated in PA than unfunctionalized MWCNTs (Figure 7 and Figure 8). 

**Figure 9:**
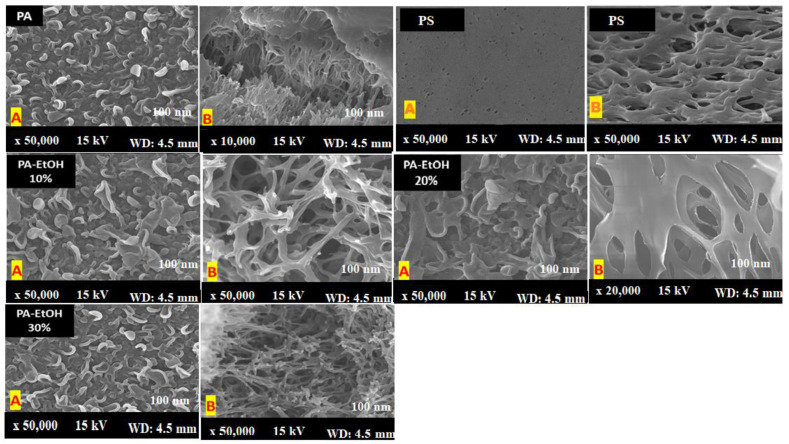
SEM images of the surfaces (**A**) and cross sections (**B**) of PA, PS, PA-EtOH 10%, PA-EtOH 20%, and PA-EtOH 30% membranes.

Furthermore, when compared to PA-MWCNTs, the carboxylic group on MWCNTs enhanced the hydrophilicity of the PA membrane [20]. An increase in the MWCNT concentration reduces the support’s mean pore size. More MPD solution is absorbed on the support when the pore size is large enough, resulting in more MPD diffusing into the hexane solution and reacting with TMC, resulting in the formation of a “leaf-like” structure [30]. Table 1 shows the findings of the direct estimation of the hydrophilicity of polyamide membranes using water contact angle data. The PA-EtOH 30% membrane was discovered to have a lower water contact angle than the PA membrane. Because of the hydrophilic –OH groups in the PA-EtOH membrane, it has higher hydrophilicity than the PA membrane, although it has a lower surface roughness. The SEM image of the cross section of membranes (Figure 9) revealed a highly porous structure, with a high degree of interconnectivity for the examined membranes. This porous structure was expected to have a positive impact on the flux rate and permeability of water. However, it can be noted that the membranes exhibit a denser morphology as the loading percentage of PA-EtOH 30% composite increases, which will probably enhance their selectivity.

### 3.5. Contact Angle Measurement

**Figure 10:**
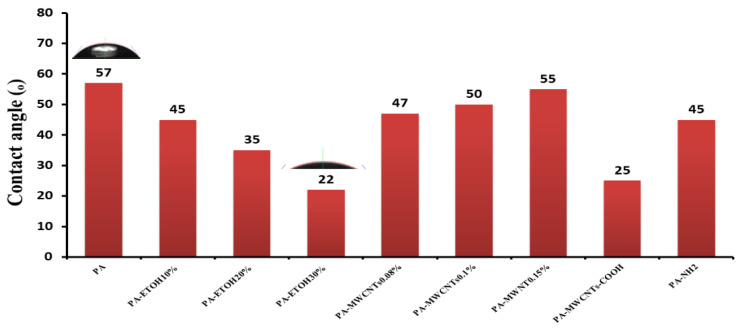
Contact angles of water droplets on the membrane’s surfaces containing PA, PA-EtOH (10%, 20%, and 30%), PA-MWCNTs (0.08%, 0.1%, and 0.15%), PA-MWCNTs-COOH, and PA-MWCNTs-NH_2_.

Hydrophilicity influences the flow and antifouling properties of membranes [31]. Contact angles of water droplets on the membrane’s surfaces containing PA, PA-EtOH (10%, 20%, and 30%), PA-MWCNTs (0.08%, 0.1%, and 0.15%), PA-MWCNTs-COOH, and PA-MWCNTs-NH_2_ were measured to determine the hydrophilicity of the membranes. The measured contact angles for the fabricated membranes are displayed in Table 1 and Figure 10. The developed membranes possess a contact angle of less than 90°, which indicates their hydrophilicity and a lower likelihood of fouling in water treatment. This was attributed to water molecules hydrating the membrane surface, preventing foulants from interacting directly with the membrane surface. Because of its hydrophilicity, the surface has a strong polarity, which attracts polar molecules like water. Permeation does not require any additional pressure. This could explain why the hydrophilic surfaces of the PA-MWCNTs-COOH and PA-EtOH membranes are greater than PA’s, and why both membranes have a higher hydrophilicity surface than the PA due to the existence of hydrophilic groups such as COOH and OH in the surface of PA membrane that promote hydrophilicity. The contact angles of the modified membranes were all reduced as a result of the large number of hydrophilic groups on the membrane surface, which contributed to boosting the membrane’s hydrophilicity. This was due to the aggregation of PA-MWCNTs reducing the effective surface of hydrophilic groups and the hydrophobic nature of CNTs. Additionally, because carbon nanotubes are hydrophobic, the contact angle values of PA-MWCNTs (0.08%), PA-MWCNTs (0.1%), and PA-MWCNTs (0.15%) were (47°, 50°, and 55°, respectively) as MWCNTs increased [32]. As shown in Figure 10, the contact angle of PA-MWCNTs-EtOH and PA-MWCNTs-COOH was 22° and 25°, respectively. The low values could be attributed to the fact that increasing the hydrocarbon tail of the alcohol or the carboxylic group led to more intrinsically wetting membrane surfaces, which correlates with the trend in surface forces as well [33]. 

## 4. Membrane Performance Evaluation 

### 4.1. Water Permeability

**Figure 11:**
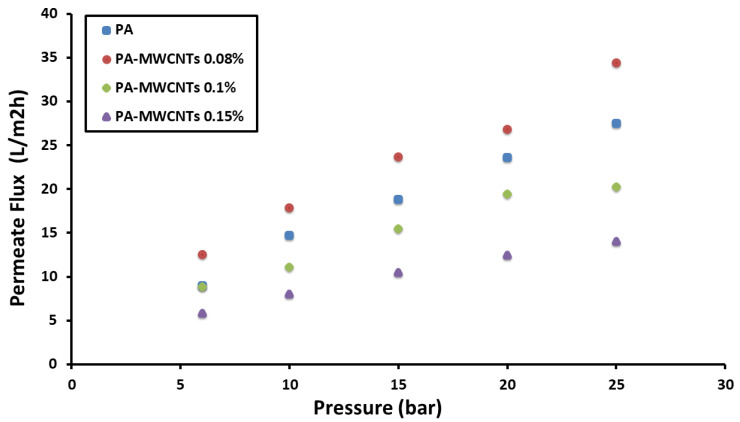
Flux rates plotted against transmembrane pressure of PA and PA-MWCNTs (0.08%, 0.1%, and 0.15%).

**Figure 12:**
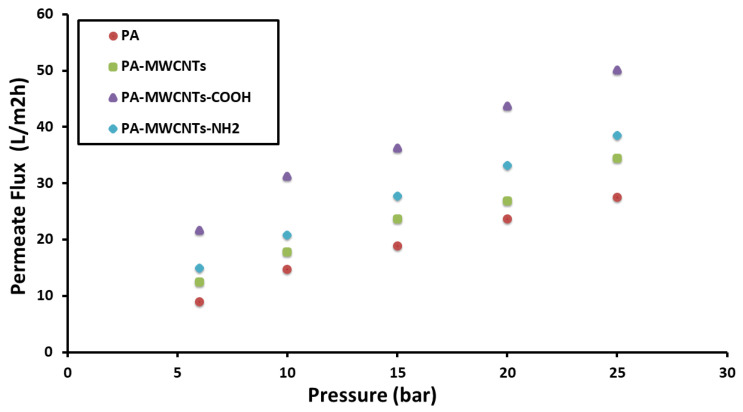
Flux rates plotted against transmembrane pressure of PA and PA-MWCNTs, PA-MWCNTs-COOH, and PA-MWCNTs-NH_2_.

**Figure 13:**
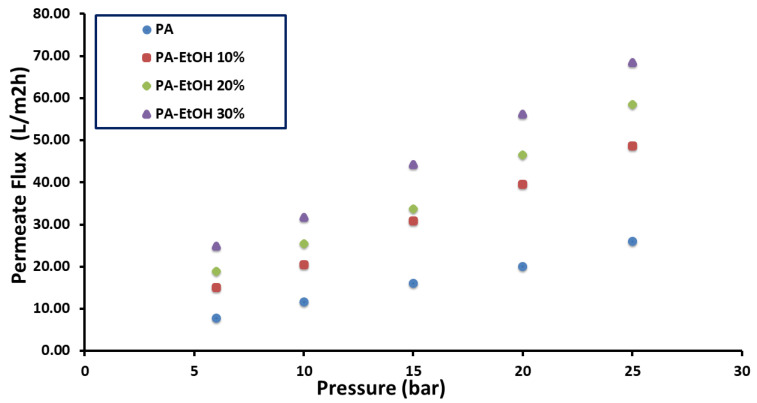
Flux rates plotted against transmembrane pressure of PA and PA-EtOH (10%, 20%, and 30%).

The hydrophilicity, surface charge, and chemical composition of the membrane had a significant impact on the water flux rate because these variables alter the interaction between the membrane surface and solution. This interaction could be mediated by a variety of secondary forces, including dipole–dipole, van der Waals, electrostatic contact, and hydrogen bonding [20]. The permeate flux was plotted versus the applied pressure (Figure 11, Figure 12 and Figure 13) for PA, PA-MWCNTs (0.08%, 0.1%, and 0.15%), PA-MWCNTs-COOH, PA-MWCNTs-NH2, and PA-EtOH (10%, 20% and 30%), respectively (Table 1). The linear regression slope between the flux and applied pressure was used to calculate the permeability results. The results showed that the RO membrane water flux in the case of the modified membrane with 30% ethanol (44.21 L/m^2^ h) increased by about 134% compared to PA (18.85 L/m^2^ h) at 15 bar.

In general, an increase in all membrane flux rates was maintained in tandem with an increase in pressure. The outcomes revealed that the RO membrane water flux in the case of the modified membrane with 30% ethanol (~44.21 L/m^2^ h) exhibited an enhancement in permeation of about 134% compared to PA (18.85 L/m^2^ h). The water flux of PA-MWCNTs-COOH (36.225 L/m^2^ h) at 15 bar, because the –COOH (polar) group, increased the hydrophilicity of the membrane surface, so the water flow of PA was increased by 85% when MWCNTs-COOH was added. PA-MWCNTs 0.08% gave the best outcome (23.662 L/m^2^ h). Accordingly, this concentration will be approved and compared with two others (0.1% and 0.15% MWCNTs). 

### 4.2. Salt Rejection 

**Figure 14:**
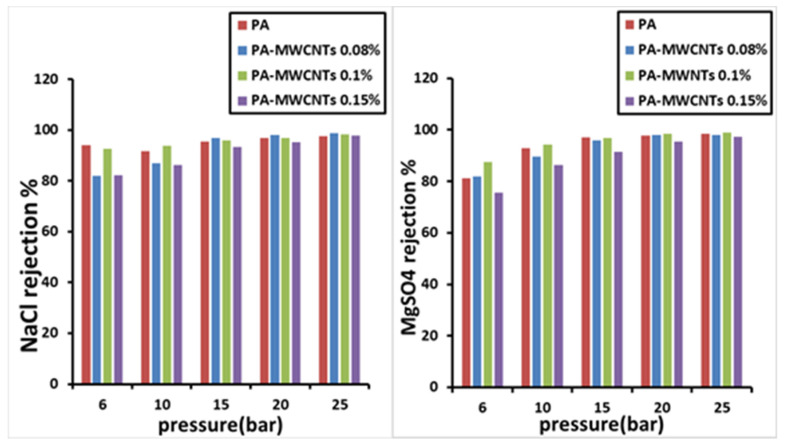
Salt rejection of PA, PA-MWCNTs (0.08%, 0.1%, and 0.15%) membranes.

**Figure 15:**
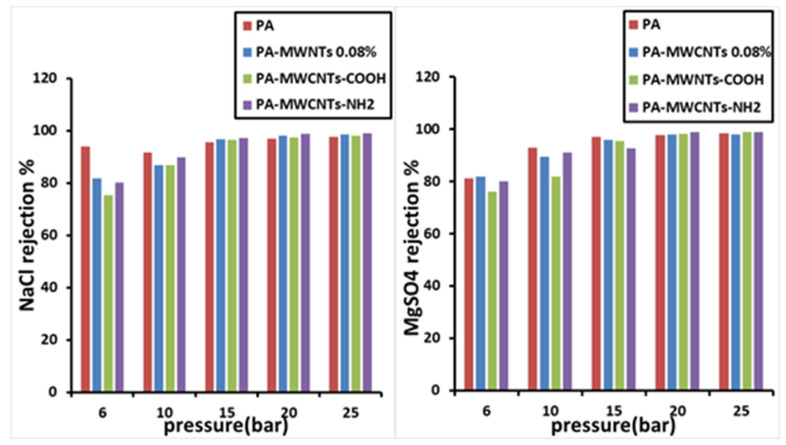
Salt rejection of PA and PA-MWCNTs, PA-MWCNTs-COOH, and PA-MWCNTs-NH_2_.

**Figure 16:**
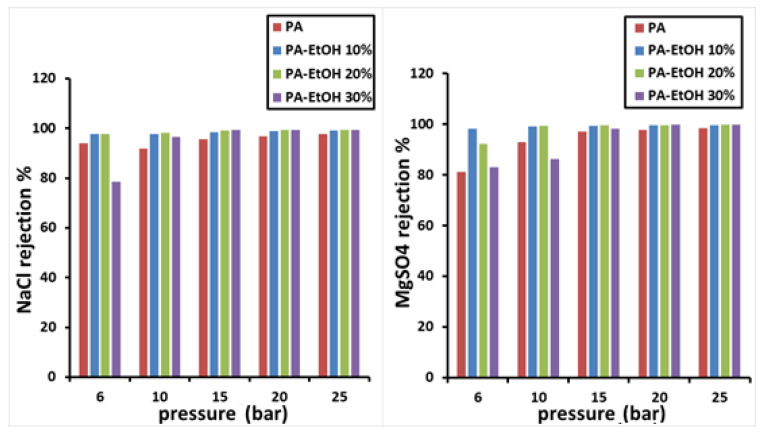
Salt rejection of PA and PA-EtOH (10%, 20%, and 30%).

The salt-rejection efficiency of all manufactured membranes was tested using a crossflow RO/NF system. The developed membranes’ salt rejection, employing PA-MWCNTs, MWCNTs (0.08%, 0.1%, and 0.15%), PA-MWCNTs-COOH, PA-MWCNTs-NH_2_, and PA-EtOH (10%, 20% and 30%), is demonstrated in Figure 14, Figure 15 and Figure 16. NaCl and MgSO_4_ were the salts rejected, with each salt solution containing 2 g/L salt. Generally, all the membranes had more than a 96% reject ratio, indicating that modified membranes can be more effective. The results showed that rejection decreased in the order of PA-EtOH 30% > PA-EtOH 20% > PA-EtOH 10% > PA-MWCNTs-NH_2_ > PA-MWCNTs-COOH > MWCNTs 0.08% > PA > MWCNTs 0.1% > MWCNTs 0.15%. In the case of decreased rejection, it appears that poor interfacial compatibility of the CNT with the polymer hampered the inclusion of CNT in the modified membrane matrix, resulting in unselective voids. Furthermore, a permeability trade-off occurred because a larger pore size was preferred over the hydrophilic CNT repulsion across the polymer matrix, leading to a permeability trade-off. This could be due to CNT obstructing the pore surface, resulting in reduced water flow through the membrane [34]. The rejection of salts for PA-EtOH 30% was 99.29% for NaCl and 99.62% for MgSO_4_ at 15 bar. This was the best result among the membranes. By inter-chain hydrogen bonds disrupting and enhancing the available free space for water permeation, combining water with alcohol improves the alcohol permeation inside the active layer. As a result, the functional groups accessible on the surface have a larger chance of contributing to the rejection of ions [35]. In this investigation, the membrane immersion period or ethanol contact time continued for 1 h. This appears to be adequate to ensure that water passes through the membranes in a controlled manner without impairing the membranes’ ability to reject salts (Table 1). Several research articles on the performance of interfacial polymerized polyamide membranes with PS pretreatment on desalination qualities through membranes based on TMC and MPD have been published. The produced membranes’ flux and salt rejection properties were compared to other membranes, as summarized in Table 2.

## 5. Conclusions

On the PS support sheet, interfacial polymerization was used to make all nine polyamide membranes. We constructed polyamide membranes containing nanopartials (MWCNTs or MWCNTs-COOH or MWCNTs-NH_2_) and treated them with ethanol to improve membrane efficiency by improving permeability toward water with high salt rejection. The PA-MWCNTs 0.15% membrane had decreased surface roughness and higher hydrophobicity according to SEM and water contact angle studies. Because of MWCNTs’ hydrophobic characteristics, increasing their loading might cause particle coagulation in a polymer matrix, limiting the membrane separation. At 15 bar, the water flux of a RO membrane modified with 30% ethanol exhibited the maximum permeate flux (68.40 L/m^2^ h) and rejection salts (99.29 percent for NaCl and 99.62 percent for MgSO_4_). This is due to PA-higher EtOH’s wettability and larger interior pores, which play a key role in accelerating the movement of water molecules. The addition of functionalized CNTs boosted the performance of the modified membranes, according to the findings. The importance of hydrophilic CNTs in improving the hydrophilicity of a modified membrane is responsible for the improvement. Hydrophilicity is produced by hydrophilic groups connected to a functionalized CNTs wall, as evidenced by the reduced contact angle of modified membranes.

## Figures and Tables

**Table 1:** Contact angle, water permeability, and salt rejection results of all membranes.

Salt Rejection at 15 bar (%)		Water Permeability at 15 bar (L m^−2^ h^−1^ bar^−1^)	Contact Angle (°)	Membrane
MgSO_4_	NaCl			
97.12	95.55	18.85	57 ± 3	PA
99.35	98.46	30.9	45 ± 5	PA-EtOH 10%
99.62	99.09	33.75	35 ± 4	PA-EtOH 20%
98.27	99.29	44.21	22 ± 4	PA-EtOH 30%
96.03	96.79	23.662	47 ± 4	PA-MWCNTs 0.08%
96.89	95.98	15.45	50 ± 3	PA-MWCNTs 0.1%
91.5	93.42	10.5	55 ± 5	PA-MWCNTs 0.15%
95.43	96.43	36.225	25 ± 2	PA-MWCNTs-COOH
92.77	97.11	27.75	45 ± 2	PA-MWNHs-NH_2_

**Table 2:** Comparison of PA-EtOH 30% membrane with previously reported membranes for salt rejection.

Membrane	Test Conditions	Flux (L/m^2^ h)	NaCl Salt Rejection %	Ref.
PA-SiO_2_ 1%	15 bar, 2000 ppm NaCl solutions	47.9	98.9%	[36]
PA-DMF 12%	16 bar, 2000 ppm NaCl solutions	3.98	98.8%	[37]
PA-NMP 82%	15 bar, 2000 ppm NaCl solutions	36	95%	[29]
PA-EtOH 30%	15 bar, 2000 ppm NaCl solutions	44.21	99.29%	This work

DMF = dimethyl formamide. NMP = 1-Methyl-2-pyrrolidone.

## Data Availability

The outcomes of this study have been included within the text.
